# Nitrogen supply enhances the physiological resistance of Chinese fir plantlets under polyethylene glycol (PEG)-induced drought stress

**DOI:** 10.1038/s41598-020-64161-7

**Published:** 2020-05-05

**Authors:** Shubin Li, Lili Zhou, Shalom Daniel Addo-Danso, Guochang Ding, Min Sun, Sipan Wu, Sizu Lin

**Affiliations:** 1 Forestry College, Fujian Agriculture and Forestry University, Fuzhou, 350002 P.R. China; 2 Chinese Fir Engineering Technology Research Center of the State Forestry and Grassland Administration, Fuzhou, 350002 P.R. China; 3 Institute of Oceanography, Minjiang University, Fuzhou, 350108 P.R. China; 4 Forest and Climate Change Division, CSIR-Forestry Research Institute of Ghana, P.O. Box UP 63, KNUST, Kumasi, Ghana; 5 College of Arts College of Landscape Architecture, Fujian Agriculture and Forestry University, Fuzhou, 350002 P.R. China; 6 Forest Park Engineering Research Center of State Forestry Administration, Fuzhou, 350002 P.R. China; 7 Polaris Education Agency, Linyi, 276000 P.R. China

**Keywords:** Ecology, Drought

## Abstract

Water and nitrogen stresses are major constraints for agricultural and forest productivity. Although the effects of water scarcity or nitrogen stress on plant growth, physiology, and yield have been widely studied, few studies have assessed the combined effects of both stresses. In the present study, we investigated the effects of different nitrogen forms (NO_3_^−^N, NH_4_^+^-N, and a combination of NO_3_^−^N + NH_4_^+^-N) on antioxidant enzyme activity, osmotic regulatory substances, and nitrogen assimilation in Chinese fir (*Cunninghamia lanceolata*) plantlets under drought stress (induced by 10% polyethylene glycol). We found that different N ionic forms had different effects on drought-stressed plantlets. Nitrogen supply greatly increased the activities of superoxide dismutase (SOD), peroxidase (POD) and polyphenol oxidase (PPO) when plantlets were exposed to water stress. The malondialdehyde (MDA) contents significantly decreased under the NH_4_^+^ + water stress treatment. The proline (Pr) contents significantly increased in both the NO_3_^−^N and NH_4_^+^-N + water stress treatment. The nitrate reductase (NR) increased by 7.1% in the NO_3_^−^ + water stress treatment, and the glutamine synthetase (GS), and the glutamate synthase (GOGAT) activity increased in all the nitrogen + water stress treatments. These results suggested that nitrogen supply could alleviate the adverse effects of drought stress on plants by enhancing antioxidant defense and improving nitrogen assimilation, while the effects on plant tolerance to drought stress varied with nitrogen ionic forms.

## Introduction

Drought is considered to be one of the most devastating threats to agriculture and forestry. As a result of economic development and climate change, many countries now face water scarcity and water pollution^1^. Drought stress hampers plant growth and physiology, biochemical processes, and productivity^2^. Plants respond to oxidative stress caused by water deficit by overproducing reactive oxygen species (ROS), which result in damage to biological molecules and cellular organelles^3,4^. Plant tolerance to abiotic stress depends largely on their tolerance to oxidative stress, i.e., the strength of their antioxidant system (SOD, POD, PPO, CAT, etc.)^5^. For example, Zhang *et al*.^6^ reported that the antioxidant enzyme activity in the leaves of drought-hardened potato (*Solanum tuberosum* L. ‘Atlantic’) seedlings was markedly higher for 7 days compared to control seedlings, but then decreased over time (7–14 days). Besides the active oxygen scavenging system, another important mechanism to adapt to drought involves an increase in osmotic adjustment compounds, such as proline, soluble sugars, and abscisic acid content^7^. Therefore, plants show a natural ability to reduce ROS accumulation and maintain the stability of the membrane system and thus alleviate the damage caused by drought stress.

Furthermore, nitrogen metabolism is crucial for drought tolerance, namely, ion uptake, nitrogen assimilation, amino acid, and protein synthesis. Nitrate (NO_3_^−^) assimilation involves the conversion of NO_3_^−^ into nitrogen dioxide (NO_2_^−^) and then into ammonium (NH_4_^+^) through nitrate reductase (NR) in the cytosol and nitrite reductase (NiR) in the chloroplasts. A nitrate reductase is the first enzyme in nitrate assimilation and its activity levels have been shown to decrease in the leaves of many species under water stress^8^. Ammonium derived from the primary nitrate reduction as well as other metabolic pathways, such as root uptake, photorespiration, and amino acid catabolism, is first converted to glutamine by glutamine synthetase (GS), and then to glutamate by glutamate synthase (GOGAT)^9^. The sequential reactions of GS and GOGAT constitute the principle pathway for assimilating ammonia (NH_4_^+^). The capacity for nitrogen metabolism, expressed by NR, GS, GOGAT etc., has been recognized as an indicator of drought tolerance in plants^10,11^. However, the effects of nitrogen supply through different nitrogen forms on the N assimilation response under drought stress are not yet well understood.

Chinese fir [*Cunninghamia lanceolata* (Lamb.) Hook.] is one of the most important evergreen conifer species in southern China, with a planting area of over 11 million ha that occupy 15.8% of forest plantations in China and 6.5% globally^12,13^. This species is distributed across 17 provinces and autonomous regions, with a large geographical range from 21°31′ to 34°03′ N, and from 101°30′ to 121°53′ E. Chinese fir has suffered the effects of drought caused by the spatial and seasonal inhomogeneity of precipitation occurring frequently due to global climate change, especially associated with the subtropical high pressure in the Pacific Ocean. Drought stress usually occurs in summer and autumn, when Chinese fir is in the fast-growing period; thus, the effects of drought on the growth and survival of Chinese fir species can be devastating^14^. Lin^15^ reported that the survival rate, current annual increment of ground diameter, and current annual increment of tree height of 1-year-old Chinese fir plantations decreased by 11.5%, 54.3%, and 36.4%, respectively, under drought conditions compared to normal precipitation conditions in 2000–2002.

Nitrogen is the most limiting nutrient for tree growth and productivity; further, NH_4_^+^ and NO_3_^−^ are the main inorganic nitrogen forms that can be absorbed and utilized by plants^16^. Currently, the declining timber yield and soil degradation of Chinese fir plantations are serious issues due to many inappropriate silvicultural practices, such as monoculture, short rotations, clear cutting, etc.^17,18^. These practices have resulted in soil nutrient depletion; and nitrogen deficiency is an especially important factor that severely limits sustainable productivity of Chinese fir plantations^19^. Until now, only a few studies have been conducted on the effects of either drought or nitrogen stress on Chinese fir antioxidant defense system or osmotic adjustment substances. For example, previous studies showed that free radicals or ROS, such as H_2_O_2_, O_2_^−^, and OH^−^, accumulated under drought stress, while CAT content increased and POD and SOD content decreased for Chinese fir species^20,21^. Li *et al*.^22^ reported that exogenous Ca^2+^ and ascorbic acid could reduce MDA content and protect the plasma membrane system from damage by abiotic stress. They also found that exogenous Ca^2+^ and ascorbic acid could increase the content of osmotic-adjustment compounds like soluble sugars and soluble proteins for Chinese fir species. However, information on the response of nitrogen-metabolism regulation in Chinese fir under drought conditions is scarce. Nitrogen addition affects plant physiological features by reducing MDA, increasing foliar free proline, and influencing antioxidant enzymes^23,24^. Ma *et al*.^25^ found that, as nitrogen stress increased, the NR activity and the light saturation point decreased among different Chinese fir clones. Although many studies have assessed physical responses of Chinese fir to either drought or nitrogen stress, the effects of addition of different nitrogen forms on the response of physiological characteristics to drought stress have not been thoroughly investigated. Furthermore, the responses to drought stress through altered nitrogen metabolism (nitrate and ammonia assimilation) have been scarcely reported. Thus, the physiological mechanisms of coniferous tree species in response to the combined effects of drought stress and nitrogen supply have not yet been clarified.

In view of this, we hypothesized that nitrogen supply would (1) increase antioxidant enzyme activity and the content of osmotic-adjustment-substances, and (2) activate nitrogen assimilation enzymes to enhance drought tolerance. To verify these hypotheses, we measured the MDA content and activities of the plant antioxidant enzyme system (SOD, POD, PPO), osmotic adjustment substances (proline, soluble sugars), and nitrogen assimilation activity (NR, GS, GOGAT) in Chinese fir needles under the supply of various nitrogen forms, (three types: NO_3_^−^N, NH_4_^+^-N, and a combination of both) and polyethylene glycol (PEG)-induced drought stress. The results of this study can provide scientific basis for drought tolerance evaluation and improvement of nutrient utilization efficiency (NUE) for Chinese fir species.

## Results

### Effects of nitrogen forms and drought stress on antioxidant enzyme activity and lipid peroxidation

**Figure 1:**
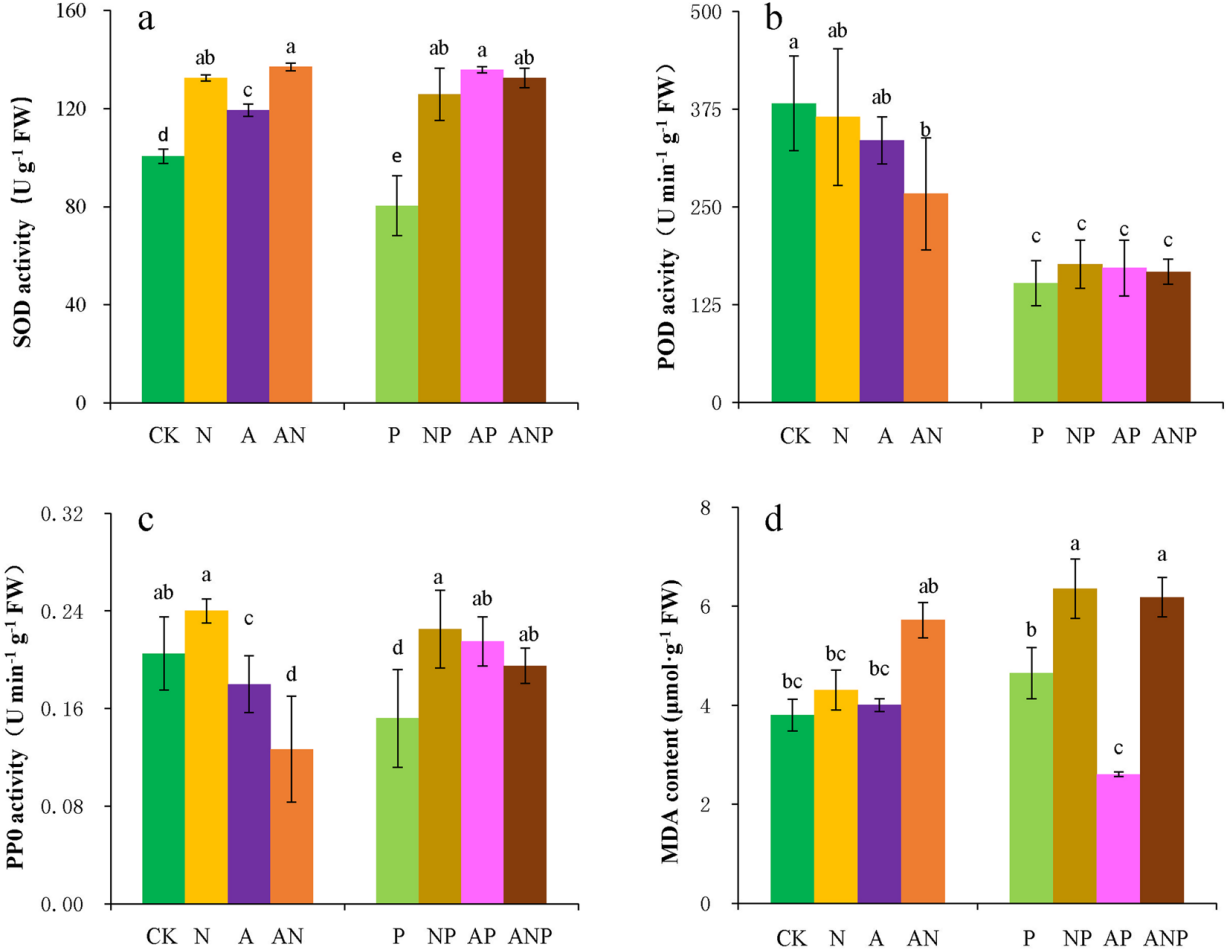
Effects of different nitrogen ion forms and water stress on SOD (**a**), POD (**b**), PPO (**c**) and MDA (**d**). Treatments: CK, seedlings treated normal water and basic nutrient without nitrogen; N, seedlings treated with NO_3_^−^N only; A, seedlings treated with NO_4_^+^-N only; AN, seedlings treated with NO_3_^−^N and NO_4_^+^-N in combination; P, seedlings with 10% PEG; NP, seedlings with NO_3_^−^N + 10% PEG; AP, seedlings with NO_4_^+^-N + 10% PEG; ANP, seedlings treated with NO_3_^−^N and NO_4_^+^-N in combination +10% PEG. Mean (± SD) was calculated for three replicates for each treatment. Vertical bars with different lowercase letters are significant at *P* < 0.05, determined by LSD test.

Under non-limiting water conditions, the SOD activity was significantly higher in Chinese fir plantlets under different nitrogen treatment, while the SOD activity was significantly lower by 20.0% under drought stress than in the control. However, the SOD activity was significantly higher by 56.5%, 68.7% and 64.8% in NP, AP and ANP, respectively (Fig. 1a) than under the water stress conditions. Under common water conditions, the POD activity decreased by 4.6%, 12.4%, and 30.3% in the NO_3_^−^N, NO_4_^+^-N, and NO_3_^−^N and NO_4_^+^-N combination conditions, respectively. The POD activity was also considerably lower under drought stress than in the control. However, the POD activity was slightly higher than that of the control treatment, by an average of 12.8%, under nitrogen treatment (Fig. 1b). The PPO activity increased by 17.1%% in the NO_3_^−^N treatment, and decreased by 12.2% and 38.2% in NO_4_^+^-N and the NO_3_^−^N and NO_4_^+^-N combination treatments, respectively. The PPO activity was also significantly lower, by 25.9%, under drought stress, while PPO activity significantly higher by 48.0%, 41.5% and 28.3%, respectively, under the NP, AP and ANP treatments (Fig. 1c).

Under non-limiting water conditions, MDA content was all higher in the NO_3_^−^N and NO_4_^+^-N combination treatment than in the control. Water stress led to a 22.4% higher MDA content in Chinese leaf under drought stress (Figs. 1d) and 36.6% and 32.9% higher content in AP and ANP treatments than in the control, while a noteworthy significant decrement was recorded at 44.0% under the AP treatment than under drought stress. Thus, different nitrogen applications increased the activities of SOD, POD, and PPO to different degrees, and decreased MDA content under NO_4_^+^-N + water stress with respect to that under drought stress.

### Effects of nitrogen forms and drought stress on osmotic adjustment substances

**Figure 2:**
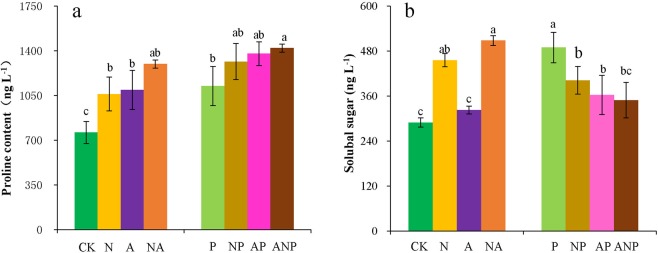
Effects of different nitrogen ion forms and water stress on Pro content (**a**) and soluble sugar content (**b**). Treatments: CK, seedlings treated normal water and basic nutrient without nitrogen; N, seedlings treated with NO_3_^−^N only; A, seedlings treated with NO_4_^+^-N only; AN, seedlings treated with NO_3_^−^N and NO_4_^+^-N in combination; P, seedlings with 10% PEG; NP, seedlings with NO_3_^−^N + 10% PEG; AP, seedlings with NO_4_^+^-N + 10% PEG; ANP, seedlings with NO_3_^−^N and NO_4_^+^-N in combination +10% PEG. Mean (±SD) was calculated for three replicates for each treatment. Vertical bars with different lowercase letters are significant at P < 0.05, determined by LSD test.

Under normal water conditions, the leaf proline contents and the sugar contents were all higher following nitrogen applications than for non-nitrogen treatments (Fig. 2). Specifically, the leaf proline contents under the NO_3_^−^N, NH_4_^+^-N, and combination treatments increased by 39.4%, 43.8% and 70.6%, respectively (Fig. 2a). The leaf sugar contents were significantly higher by 57.3% and 75.4% respectively under the NO_3_^−^N and combination treatments than under CK (Fig. 2b). Similarly, the leaf proline contents and the sugar contents of drought-stressed seedlings were considerably higher, by 47.8% and 69.0%, respectively, than in non-stressed control plants. However, the proline contents were higher by 17.0%, 22.6%, and 26.5% in NP, AP, and ANP treatments, respectively, than that in drought-stressed seedlings, while the sugar contents were significantly lower by 17.9%, 25.8%, and 28.6%, respectively.

### Effects of nitrogen forms and drought stress on nitrogen reduction and assimilation

**Figure 3:**
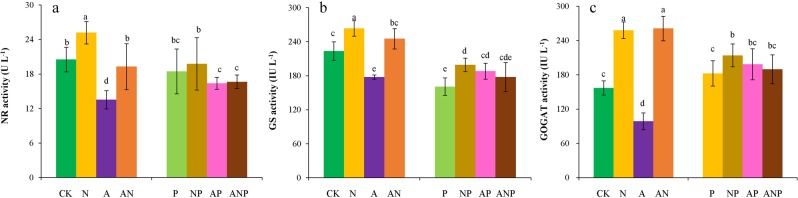
Effects of different nitrogen ion forms and water stress on NR activity (**a**) and GS activity (**b**) and GOGAT activity (**c**). Treatments: CK, seedlings treated with normal water and basic nutrient without nitrogen; N, seedlings treated with NO_3_^−^N only; A, seedlings treated with NO_4_^+^-N only; AN, seedlings treated with NO_3_^−^N and NO_4_^+^-N in combination; P, seedlings with 10% PEG; NP, seedlings with NO_3_^−^N + 10% PEG; AP, seedlings with NO_4_^+^-N + 10% PEG; ANP, seedlings with NO_3_^−^N and NO_4_^+^-N in combination +10% PEG. Mean (±SD) was calculated for three replicates for each treatment. Vertical bars with different lowercase letters are significant at P < 0.05, determined by LSD test.

Under normal water conditions, the NR activity was significantly higher in the NO_3_^−^N treatment and lower in NH_4_^+^-N treatment. Compared to that under CK, the NR activity was lower by 10.1% under water stress. However, NO_3_^−^N treatment led to a 7.1% higher NR activity under NP treatment than under water stress (Fig. 3a). The same trend was observed for the GS activity, which was significantly higher by 17.9% in the NO_3_^−^N treatment and significantly lower by 20.5% in the NH_4_^+^-N treatment respectively than under CK. The GS activity was significantly higher by 23.8%, 16.9%, and 10.4% for NP, AP, and ANP treatments, respectively, than under water stress (Fig. 3b). In contrast, under normal water conditions, the GOGAT activity was significantly higher by 64.4% and 66.4% in the NO_3_^−^N and the combination treatments, respectively, than CK. the GOGAT activity was slightly higher under water stress than under normal water conditions. Compared to water stress, the GOGAT activity was higher by 17.2%, 8.8% and 3.9% under the NP, AP, and ANP treatment, respectively, and the differences were significant under NP treatment. Therefore, nitrogen supply, specifically the NO_3_^−^N ion, can alleviate the negative effects of drought on nitrate reduction and nitrogen assimilation in plants.

## Discussion

We confirmed our first hypothesis that nitrogen supply would enhance antioxidant enzyme activity and osmotic adjustment by inducing proline accumulation to alleviate the physiological damage caused by drought stress (Figs. 1a–c and 2a). However, the effects of nitrogen varied with the nitrogen ionic form supplied. Under drought stress, plants face the risk of ROS toxicity, which may result in lipid peroxidation and oxidative damage to DNA^26^. Recently, it was proposed that plants have an effective ROS scavenging and signaling system, as suggested by a correlation between drought tolerance and antioxidant enzyme activities^27^. We found that different nitrogen ionic forms had different effects on antioxidant enzyme activities. Needle SOD activity and PPO activity significantly improved under nitrogen + drought stress treatment, but the extent of the increase varied with the nitrogen ion form supplied (Fig. 1a,c). Needle POD activity also showed higher values by supplying nitrogen under drought stress (Fig. 1b). While MDA content of Chinese fir needles decreased significantly by 44.0% under NH_4_^+^-N + drought stress (Fig. 1d). These findings indicate that nitrogen supply reduced oxidative damage to Chinese fir plantlets under drought stress; which are consistent with those of previous studies. For example, Gou *et al*.^28^ reported that foliar-applied urea could enhance SOD and POD activities and decrease MDA content of Maize (*Zea mays* L.) under drought conditions. Saneoka *et al*.^29^ demonstrated that high nitrogen fertilizer rates (100 and 200 kg/ha) prevented cell membrane damage and enhanced osmotic regulation in *Agrostis palustris* Huds. under water stress. Nutrients play important role in decreasing the adverse effects of drought in plants, through maintaining enzyme activity, charge balance and providing osmoticum. Wu *et al*.^30^ found the antioxidant activities such as SOD and catalase (CAT) were significantly enhanced, but MDA content was remarkably reduced by supplying zinc nutrient for cotton (*Gossypium Hirsutum*) under drought stress, which is consistent with our results.

Besides this oxygen scavenging system, the amount of osmotic adjustment compounds, such as proline, soluble sugars, and starch, may increase to improve plant tolerance to drought stress^30^. Some studies have reported that proline and soluble sugar, synthesized during photosynthesis, play an important role in osmoregulation, and that they increase in response to water deficits^31,32^. Furthermore, low molecular substances, such as soluble sugars, were superior to macromolecular starch in osmoregulation in response to drought stress^33^. In this study, proline content significantly increased in the NO_3_^−^N and NH_4_^+^-N + combination treatment under drought stress, while sugar content decreased in nitrogen + drought stress treatment, compared to the water stress treatments (Fig. 2a,b). Several studies have reported similar results on the effects of N supply on osmotic adjustment in response to water stress. For example, Premachandra *et al*.^34^ reported that solute concentrations (e.g., sugars and K^+^) greatly influenced the osmotic potential at a higher nitrogen application rate (ammonium sulfate, 300 kg ha^-1^) in soybean (*Glycine max* L.). Similarly, Saneoka *et al*.^29^ found that although osmotic potential may decrease under water stress, osmotic adjustment increased with increasing N supply level in *A. palustris*, thereby maintaining the water potential gradient that drives water flow vertically (upwards) and horizontally. However, Villar-Salvador *et al*.^35^ observed that high N fertilization decreased plasmalemma stability and favored higher water potential, while drought hardening increased plasmalemma stability and increased tissue non-structural carbohydrates and N concentration. They concluded that drought-hardening and N fertilization exert different effects on the physiological stress tolerance of *Pinus* seedlings, with drought hardening increasing stress tolerance by inducing osmotic adjustment and triggering the scavenging system, and N nutrition reducing the extent of the damaging effects by directly and indirectly promoting a wide array of biochemical processes.

The NR is the first enzyme to assimilate NO_3_^−^ to NH_4_^+^. After NH_4_^+^ or NO_3_^−^, or both, are absorbed by roots, a large amount of NO_3_^−^ is assimilated^36^. Thus, most NO_3_^−^ is converted into NH_4_^+^ by NR, and NH_4_^+^ is locally assimilated to glutamine and glutamate via GS and GOGAT^37^, while the remaining ions are translocated to the leaves or other organs. Drought stress influences the activity of these N metabolism enzymes^38,39^. In our study, the NR activity was higher by 10.1% in NO_3_^−^N + water stress than that under water stress only (Fig. 3a). GS and GOGAT activity in Chinese fir needles also increased to varying degrees under nitrogen + water stress with the enhancement observed in the order NO_3_^−^N + water stress> NH_4_^+^-N + water stress> the NO_3_^−^N and NH_4_^+^-N combination treatment + water stress (Fig. 3b,c). Our study suggested that increased NO_3_^−^N supply contributed to the conversion of NO_3_^−^ to NH_4_^+^ and increased NH_4_^+^ assimilation, which is consistent with the findings of previous studies. Meng *et al*.^38^ found that drought stress decreased NR and GOGAT activities in the leaves of *Populus simonii* seedlings, but NR activity increased in response to normal nitrogen supply (1 m*M* NH_4_NO_3_) under drought stress. These authors found that two ammonium transporter genes (*AMTI; 2* and *AMTI; 6*), closely related to NH_4_^+^ uptake, were up-regulated in response to drought stress. Zaghdoud *et al*.^40^ reported that NO_3_^−^ or NO_3_^−^/NH_4_^+^ co-provision alleviated the effect of salt stress regarding water balance in broccoli plants through an enhanced rate of photosynthesis and an improvement of N metabolism (NR and GS). However, Silveira *et al*.^41^ found a rapid increase in nitrate content in roots and a marked reduction in leaf NR activity in cowpea plants (*Vigna unguiculata* L.) under both water stress and NO_3_^−^ supply (5 m*M*). In conclusion, the effects of nitrogen ionic forms (i.e., NO_3_^−^, NH_4_^+^ and NO_3_^−^/NH_4_^+^) on plant tolerance to drought stress may vary among species and the parameters that are consideration.

## Conclusion

Generally, plants that are adapted to low pH tend to take up nitrogen in the form of ammonium (NO_4_^+^) or amino acids, whereas plants that are adapted to high pH and highly aerobic soils prefer nitrate (NO_3_^−^) uptake. Soil nitrogen deficiency and water stress are both important factors that restrict the sustainable development of Chinese fir plantations. Different nitrogen ion forms had differing effects on the enhancement of POD, SOD, PPO, GS and GOGAT activity. NH_4_^+^ led to significantly lower MDA content while NO_3_^−^ led to slightly higher NR activity than that in water-stressed plantlets. Proline content significantly increased under the NO_3_^−^N and NH_4_^+^-N combination treatment. In summary, the addition of nitrogen greatly decreased the negative effects of drought stress and enhanced the drought tolerance of Chinese fir seedlings, but the enhancement effects of nitrogen varied with ion forms.

## Materials and methods

### Plant materials and applied treatments

The original plant materials were obtained from the seeds of the third-generation seed-orchard of Chinese fir species in Youxi National Farm, Fujian province. The seeds of each available genotype were planted in pots and the seedlings growth were evaluated. One superior Chinese fir clone (No. 7–14, propagated asexually) was chosen as the study material. Besides its growth rate, the No. 7–14 family has strong drought resistance and nitrogen absorption ability^42^. The average seedling height was 38.5 cm. Plantlets were grown using a water cultivation method under controlled conditions (16:8 h light: dark regime, 120 μmol m^−2^ s^−1^ photon flux; at 25 °C and 60% RH) in a growth chamber (LT-ACC400, China).

The basic nutrient solution was controlled using a modified Hoagland nutrient solution that contained K_2_SO_4_ (0.41 g L^−1^), Mg_2_SO_4_·7H_2_O (0.49 g L^−1^), KH_2_PO_4_ (0.136 g L^−1^); H_3_BO_3_ (0.286 g L^−1^), H_2_MoO_4_ (0.0623 g L^−1^), MnCl_2_·4H_2_O (0.181 g L^−1^), CuSO_4_·5H_2_O (0.008 g L^−1^), ZnSO_4_·7H_2_O (0.022 g L^−1^); ferric salts, FeSO_4_·7H_2_O (0.278 g L^−1^), and EDTA-Na_2_ (0.373 g L^−1^). Three nitrogen sources were used to provide 4.571 m*M*: NO_3_^−^N from Ca(NO_3_)_2_ at 0.3748 g L^−1^, NH_4_^+^-N from (NH_4_)_2_SO_4_ at 0.3016 g L^−1^, and the combination treatment using NO_3_^−^N and NH_4_^+^-N from Ca(NO_3_)_2_ at 0.1874 g L^−1^ and (NH_4_)_2_SO_4_ at 0.1508 g L^−1^. We then added nitrification inhibitor (0.01 m*M* Dicyandiamide, DCD) to each of these nutrient solutions. In total, eight treatments were applied: CK (normal water + basic nutrient solution without nitrogen), N (NO_3_^−^N only), A (NH_4_^+^-N only), AN (NO_3_^−^N + NH_4_^+^-N), P (10% PEG), NP (NO_3_^−^N + 10% PEG), AP (NH_4_^+^-N + 10% PEG), and ANP (NO_3_^−^N + NH_4_^+^-N + 10% PEG). After acclimation, plantlets were divided into eight treatment groups; each with three replicates.

### Antioxidant enzyme activity and lipid peroxidation determination

Enzyme liquid extraction: Fresh one-year needles (~0.2 g) were homogenized in liquid nitrogen in centrifuge tubes (5 mL) for 15 min, and then phosphate buffer (4 mL, pH = 7) was added. The homogenates were centrifuged at 10,000 *g* for 20 min at 4 °C. The supernatants were used to determine the antioxidant enzyme activity as described below.

Superoxide dismutase activity (SOD; EC 1.15.1.1): SOD was determined by the inhibition of the photochemical reduction of nitroblue tetrazolium (NBT) according to Costa *et al*.^42^. The reaction mixture contained 0.1 mL of enzyme extract in 50 m*M* potassium phosphate buffer, (pH = 7.8), 0.1 m*M* EDTA, 50 m*M* methionine, 75 μ*M* NBT, and 20 μ*M* riboflavin. The reaction mixtures were placed under a high intensity lamp (4000 lx) for 15 min. They were then placed in the dark to stop the reaction, and the absorbance at 560 nm was recorded (Puxi Instrument, Beijing, China). One unit of SOD was defined as the amount of enzymes required to produce 50% inhibition of NBT reduction.

Peroxidase activity (POD; EC 1.11.1.7): the POD activity was determined according to Ekmekca and Terzioglu^43^. The reaction mixture contained 2.9 mL of 50 m*M* potassium phosphate buffer (pH = 6.0), 1 mL of 50 m*M* guaiacol, 1 mL of 50 m*M* H_2_O_2_, and 0.1 mL of enzyme extract. Absorbance at 470 nm was recorded.

Polyphenol oxidase activity (PPO; EC 1.14.18.1): the PPO activity was determined at 420 nm in a spectrophotometer^44^. The reaction mixture contained 1.5 mL of 0.02 M NaSO_4_ solution, 5 m*M* substrate, and 1.5 mL of enzyme extract. After the reaction was completed, absorbance at 420 nm was recorded every 1 min. In total, five absorbance readings were recorded. One unit of enzyme activity was defined as the amount of enzymes required to cause a rate of change of 0.001 absorption unites per min at 420 nm.

Lipid peroxidation determination: Lipid peroxidation was estimated by measurement of malondialdehyde (MDA, a product of lipid peroxidation) using thiobarbituric acid (TBA) according to Hasanuzzaman *et al*.^45^. The reaction mixture was homogenized in a centrifuge tube containing 2.5 mL of 0.5% TBA and 1.5 mL of enzyme extract. The mixture was placed in boiling water for 15 min and centrifuged for 10 min at 4000 rpm after cooling. The supernatant was measured and absorbance recorded at 450 nm, 532 nm, and 600 nm. Results were expressed as μmol g^-1^ on a fresh weight (FW) basis.

### Osmotic adjustment substances

Enzyme liquid extraction: Chinese fir needle samples (0.1 g) were ground in liquid nitrogen and dissolved in 0.9 mL phosphate buffer (pH = 7.2). The mixtures were centrifuged for 10 min at 10,000 *g*, and the supernatants used for determination of osmotic-adjustment substances.

Proline content: Proline was measured according to Bates *et al*.^46^. Briefly, the supernatant was mixed with acid ninhydrin with glacial acetic acid and phosphoric acid. This mixture was incubated in a boiling-water bath for 1 h. Cooling toluene was then added. After chromophore containing toluene was produced, absorbance was read at 520 nm.

Soluble sugars: Soluble sugars were determined by the anthrone method^47^. Reaction mixtures contained 1 mL extract, 1 mL distilled water, 0.5 mL mixed reagent (1 g anthrone + 50 mL ethyl acetate), and 5 mL H_2_SO_4_ (98%). The mixtures were heated in a boiling-water bath for 1 min. After cooling, absorbance was recorded at 630 nm.

### Nitrogen metabolism

Nitrate reductase activity (EC 1.6.6.1): the NR activity was determined according to Silveira *et al*.^41^. Samples (0.2 g) of 7 mm length were placed in vials of ice – cold incubation medium, consisting of 100 m*M* K-phosphate buffer (pH 7.5), 50 m*M* KNO_3_, and 1% (v/v) isopropanol. Tissues were vacuum-infiltrated for 2 min at -67 kPa, and then incubated in water in the dark for 30 min at 30 °C. After incubation, the concentration of nitrite released into the medium was determined by measuring absorbance at 540 nm.

GS activity (EC 6.3.1.2): Enzyme liquid extraction was performed following the method for osmotic adjustment substances. GS activity was determined by the hydroxamate biosynthetic method with the following reaction mixture: Tris-HCl buffer (pH = 7.0), 200 μL 300 m*M* sodium glutamate (pH = 7.0), 200 μL 30 m*M* ATP (pH = 7.0), 200 μL 500 m*M* MgSO_4_, and 200 μL 1000 m*M* hydroxylamine hydrochloride neutralized with 1000 m*M* HCl and 500 μL of enzyme extract. The mixtures were incubated at 30 °C for 30 min. After brown complex formation, absorbance was recorded at 540 nm.

GOGAT activity (EC 1.4.7.1): The mixture contained 25 m*M* Tris-HCl buffer (pH 7.6), 0.4 mL 20 m*M* L-glutamine, 0.05 mL 0.1 *M* 2-oxoglutarate, 0.1 mL 10 m*M* KCl, 0.2 mL 3 m*M* NADH, and 0.5 mL of enzyme extract. Absorbance was read at 340 nm.

### Statistical analysis

One-way analysis of variance (ANOVA) was performed to determine significant treatment effects, followed by the least significant difference test (LSD) for separate the means. The data are means ± SE. Differences at *P* ≤ 0.05 were regarded as significant. The software SPSS Statistical Package (SPSS 12.0, SPSS Ins., IL, USA) was used to perform the statistical analysis.
